# Prehospital portable ultrasound for safe and accurate prehospital needle thoracostomy: a pilot educational study

**DOI:** 10.1186/s13089-022-00270-w

**Published:** 2022-06-13

**Authors:** Zachary E. Dewar, Stephanie Ko, Cameron Rogers, Alexis Oropallo, Andrew Augustine, Ankitha Pamula, Christopher L. Berry

**Affiliations:** 1 Department of Emergency Medicine, Guthrie Robert Packer Hospital, One Guthrie Square, Sayre, PA 18840 USA; 2 Wilkes University, 84 West South Street, Wilkes-Barre, PA 18766 USA; 3 Greater Valley EMS, 904 North Lehigh Ave, Sayre, PA 18840 USA

**Keywords:** Pneumothorax, Tension pneumothorax, Emergency medical services, Point-of-care ultrasound, Trauma, Needle decompression, Needle thoracostomy, POCUS

## Abstract

**Background:**

Simulated needle thoracostomy (NT) using ultrasound may reduce potential injury, increase accuracy, and be as rapid to perform as the traditional landmark technique following a brief educational session. Our objective was to determine if the use of an educational session demonstrating the use of handheld ultrasound to Emergency Medical Services (EMS) staff to facilitate NT was both feasible, and an effective way of increasing the safety and efficacy of this procedure for rural EMS providers.

**Methods:**

A pre/post-educational intervention on a convenience sample of rural North American EMS paramedics and nurses. Measurement of location and estimated depth of placement of needle thoracostomy with traditional landmark technique was completed and then repeated using handheld ultrasound following a training session on thoracic ultrasound and correct placement of NT.

**Results:**

A total of 30 EMS practitioners participated. Seven were female (23.3%). There was a higher frequency of dangerous structures underlying the chosen location with the landmark technique 9/60 (15%) compared to the ultrasound technique 1/60 (1.7%) (*p* = 0.08). Mean time-to-site-selection for the landmark technique was shorter than the ultrasound technique at 10.7 s (range 3.35–45 s) vs. 19.9 s (range 7.8–50 s), respectively (*p* < 0.001). There was a lower proportion of correct location selection for the landmark technique 40/60 (66.7%) when compared to the ultrasound technique 51/60 (85%) (*p* = 0.019). With ultrasound, there was less variance between the estimated and measured depth of the pleural space with a mean difference of 0.033 cm (range 0–0.5 cm) when ultrasound was used as compared to a mean difference of 1.0375 cm (range 0–6 cm) for the landmark technique (95% CI for the difference 0.73–1.27 cm; *p* < 0.001).

**Conclusions:**

Teaching ultrasound NT was feasible in our cohort. While time-to-site-selection for ultrasound-guided NT took longer than the landmark technique, it increased safe and accurate simulated NT placement with fewer identified potential iatrogenic injuries.

**Supplementary Information:**

The online version contains supplementary material available at 10.1186/s13089-022-00270-w.

## Introduction

### Background

Of the potential interventions prehospital advanced life support (ALS) personnel may employ in the care of injured patients, needle thoracostomy (NT), or needle decompression, is one of the most potentially lifesaving and time-sensitive. NT is employed when a patient has a suspected pneumothorax with obstructive shock, or tension, pathology. This procedure involves introducing a catheter into the affected thoracic cavity to release the entrapped volume of air and is applied as a temporizing measure prior to definitive tube thoracostomy [1]. Traditionally, this is accomplished by visually or manually identifying the desired landmarks on the patient where introducing the catheter would be both safe and effective.

### Importance

NT is not without risk. Incorrect placement of NT can result in iatrogenic injury to underlying structures [1, 2] and lead to grievous complications or death. Additionally, NT may fail due to poor technique, patient body habitus, and catheter length [3–6]. Our objective, therefore, was to explore novel means by which to improve the safety and efficacy of NT.

Point-of-care ultrasonography (POCUS) has previously been utilized to address the diagnostic dilemma of detection of pneumothorax in the prehospital environment [7–12]. The basis for this prehospital usage of POCUS in the detection of pneumothorax was described first in the in-hospital setting, and early experiences noted that POCUS was both sensitive and specific in diagnosis [13–15].

Prehospital POCUS is becoming more widely adopted for its diagnostic abilities to aid in decision-making in traumatically injured patients [16–18]. Therefore, it would seem that the natural progression for the usage of POCUS in the field would be to apply it procedurally. POCUS has previously been described to improve the accuracy in tube thoracostomy placement in Emergency Medicine residents [19]. We aimed to pilot an educational study intervention training ALS prehospital personnel to use the handheld POCUS to aid in the detection of pneumothorax and apply it during the procedure to improve safety and success.

### Goals of this investigation

We hypothesized that the use of an ultrasound device would reduce potential iatrogenic injuries, increase placement accuracy, and be just as rapid to use as the landmark technique during a simulated NT following a brief educational session.

## Methods

### Study design, participant recruitment, and intervention

The study was approved by The Guthrie Clinic Institutional Review Board (IRB). It was approved for continuing medical education for the prehospital nurses and paramedics through the local EMS governance structure in the two states participating. Participants were recruited from ALS agencies which provide 911, critical care transport, and helicopter EMS services to a 13-county area in rural Pennsylvania and Upstate New York within the United States of America. Recruitment occurred over a 2-month period. Group sessions included a maximum of eight ALS providers, and subjects provided information including demographics, level of training, and years of EMS experience (see Additional file 1: Survey instrument).

Subjects received an invitation to participate, which contained the elements of informed consent. The IRB did not require a specific informed consent form due to the study’s voluntary and minimal risk nature. Prior to the education session, each provider completed a simulation where they were presented with a healthy volunteer model and given a case of a patient with a suspected tension pneumothorax. Volunteer models used varied, but generally consisted of men aged 25–35, with a body mass index (BMI) that ranged from 24 to 35 kg/m^2^. The participants were asked to mark the anatomic location for needle decompression with an ultraviolet pen and verbally state the estimated depth of needle insertion necessary for adequate decompression. This site was then recorded following each participant, with ultrasound utilized by the investigators to determine the actual selected location relative to the target rib space, the depth required to access the pleural space, and the presence of underlying structures (vasculature, heart, liver, diaphragm). The episode was timed, and subjects were randomly assigned to either a left or right-sided pneumothorax. The participants completed this two times, first with an anterior, mid-clavicular approach followed by a lateral, anterior axillary line approach. During the post-education session, the opposite side was used in the scenario.

Following the landmark simulation, a board-eligible EMS physician provided an educational intervention consisting of a one-hour lecture describing indications for needle thoracostomy, along with a review of introductory ultrasound physics, technique, and thoracoabdominal structures involved in the procedure. Lung sliding identification and the utilization of M-mode in the detection of pneumothorax were discussed. The lecture intervention was followed by a hands-on ultrasound scanning time of approximately 30 min, which allowed all participants to familiarize themselves with the device and the use of ultrasound to identify pneumothorax, as well as to detect underlying dangerous structures on live volunteer models.

A post-education simulated skill station then followed and had the participants utilize the ultrasound to find the appropriate location for both anterior and lateral needle decompression. The same data were collected, although in the post-session, the participants could derive the depth to the pleural cavity utilizing the ultrasound device.

Participants were not constrained in the way that they would use the ultrasound device during the session, and they could move the probe as they felt fit and utilize M-mode if needed. They were not given any verbal or non-verbal feedback from the observing investigators or research assistant who was timing and recording data. The participant’s total time was marked from when they indicated that they were ready to proceed and stopped when they stated that they had selected the final location for their needle placement. For the purposes of this study, the participants were not asked to complete a task-trainer-type demonstration of the needle thoracostomy skill itself. No verbal coaching, reference to materials, textbooks, or electronic resources was allowed during either landmark or ultrasound data collection.

Following the completion of both the pre-education and post-education simulated skill stations, the participants were shown a series of ten multimedia exhibits to test their interpretation of ultrasound images of the thorax. These consisted of five still images of both normal lungs and of pneumothorax, as well as five cinematic clips of thoracic ultrasounds of both normal lungs and pneumothorax. The participant’s answer to each question, and its correctness, was recorded for each of the ten total exhibits.

The handheld ultrasound device utilized in this study was a Butterfly iQ (Butterfly Network, Guilford, CT, USA), this was connected to an Apple iPad Pro Device for real-time visualization by the investigators and participants (Apple Corporation, Cupertino, CA, USA).

### Outcomes

Our primary outcome measurement was the number of potential iatrogenic injuries, and secondary outcomes included the time to complete the simulated scenario (time to the determination of site), the proportion of correct anatomical placement, and difference in measured depth compared to the estimated depth required for successful pleural space access.

### Sample size

Given the pilot nature of this educational study of a novel intervention, there was no previous data available with which to calculate the power needed. Due to the difficulty of recruiting participants from our rural area and constraints posed by the novel coronavirus pandemic, we recruited a convenience sample of 30 prehospital paramedics and nurses.

### Analysis

Results were grouped according to if the skill simulated skill attempt had been completed using the landmark or ultrasound-based technique. Descriptive statistics were calculated for demographic information. Analysis of categorical proportions was conducted with the Wald test, and testing for means was completed using Student’s *t*-tests.

All results were analyzed with IBM SPSS version 28 (Armonk, NY, USA).

## Results

A total of 30 EMS practitioners participated. Seven were female (23.3%). Twenty-five were certified at the Emergency Medical Technician Paramedic (EMT-P) level, and five were registered at the Prehospital Registered Nurse (PHRN) level. Mean years of ALS EMS experience was 15 years (range 0–44) (see Table 1).

**Table 1:** Demographic information of study participants

Participant Characteristics	Total (n = 30)
Age, median (IQR)	32 (27.45)
Sex, n (%)	
Male	23 (76%)
Female	7 (23%)
Years of Experience, median (IQR)	10 (4.18)
Cert. Level, n (%)	
EMT-P	25 (85%)
PHRN	5 (15%)
Prior U/S training, n (%)	
No	27 (90%)
Yes	3 (10%)
Critical care transport, n (%)	
No	24 (80%)
Yes	6 (20%)

*EMT-P* Emergency Medical Technician-Paramedic, *PHRN* Prehospital Registered Nurse, *U/S* Ultrasound

The mean time to complete the simulated task using the landmark technique was shorter than that of the ultrasound technique at 10.7 s (range 3.35–45 s) vs. 19.9 s (range 7.850 s), respectively (*p* < 0.001). There was a lower proportion of correct placements for the landmark technique, 40/60 (66.7%), when compared to the ultrasound technique, 51/60 (85%) (*p* = 0.019) (see Table 2).

**Table 2:** Outcome measurements by the use of ultrasound as compared to the landmark technique

	Ultrasound (n = 60)	Landmark (n=60)	*p*
Dangerous underlying structure	1/60 (1.7%)	9/60 (15%)	0.008
Time to completion, s			
Mean (SD)	19.9 (10.6)	10.7 (7.1)	< 0.001
Median (range)	17 (7.8–50)	9.21 (3.35–45)	
Correct placement*	51/60 (85%)	40/60 (66.7%)	0.019

*Defined as in the correct interspace and midline location specified for the attempt

When ultrasound was used, there was less variance between the estimated and measured depth of the pleural space with a mean difference of 0.033 cm (range 0–0.5 cm) when ultrasound was used as compared to a mean difference of 1.0375 cm (range 0–6 cm) for the landmark technique (95% CI for the difference 0.73–1.27 cm; *p* < 0.001).

**Fig. 1:**
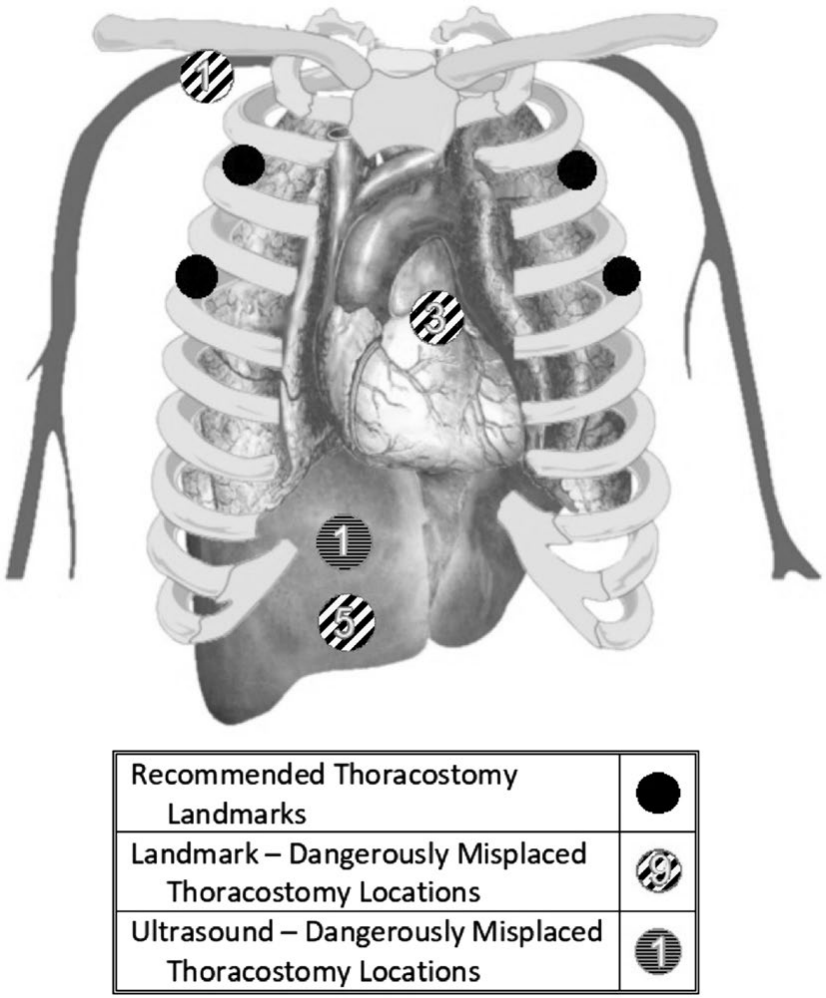
In this illustrated figure, the correct locations (as specified previously as being 2nd ICS MCL, or 4th/5th ICS AAL/MAL) are represented in black. The frequency of the presence of a dangerous underlying structure is stratified by what technique was used, with diagonal slashed circles representing landmark-based attempts, and the horizontal dashed circle representing the single ultrasound-based attempt with a dangerous underlying structure

Critical anatomic structures were more frequently in the path of the needle with the landmark technique 9/60 (15%) when compared to the ultrasound technique 1/60 (1.7%) (*p* = 0.008). The most commonly encountered underlying structure was the liver in 6/120 total skill attempts (5%), followed by the heart or mediastinal structures in 3/120 skill attempts (2.5%) (see Table 2 and Fig. 1).

Following the educational intervention and ultrasound data collection station, participants were able to identify pneumothorax correctly on multimedia stimulus using ultrasound video clips and still images 94.7% of the time (range 70–100%).

## Limitations

This study was designed to be a pilot study. Thus, it was limited to one group of EMS professionals, which was heterogeneous with respect to their previous experience level and exposure to NT. NT is a low-frequency event in our rural EMS practice. Therefore, our findings do not necessarily translate to high-volume urban EMS agencies. Importantly, in a high-volume urban environment that may perform more needle decompressions, the number of paramedics may be higher, resulting in the same low-volume skill per paramedic regardless of the paramedic’s environment. This limit to generalizability is compounded when we factor in the differences in EMS system design between the North American model, predominately staffed by paramedical practitioners, and the European model, where staffing is predominately physician-based [20]. We note the potential for an educational effect given participants reviewed anatomy during the educational session, irrespective of its focus on ultrasound. This effect was not accounted for within our study design. Furthermore, our study took place with a small group of EMS professionals using one handheld ultrasound. There is significant variability between the handheld devices currently deployed in the field. Additionally, our ultrasound models were homogenous with respect to weight, and all had BMI < 35 kg/m^2^. Finally, we were unable to include a control group that had received teaching on pneumothorax and proper placement of NT without an ultrasound component to delineate the effect of the educational intervention on the outcomes measured.

## Discussion

Our data demonstrate that a brief lecture followed by hands-on ultrasound training in an ultrasound-naive cohort of EMS providers was effective in allowing for rapid identification of appropriate needle thoracostomy position in the immediate post-education period during a simulated session in a non-clinical environment. It appeared feasible to deliver the training, and all participants were able to successfully complete the session as delivered. Ultrasound guidance resulted in fewer critical anatomic structures potentially injured by the procedure and required less than 10 s of additional time compared to the landmark technique.

Additionally, with the utilization of POCUS, there was less variation in the distance between the pleural space estimated and the actual distance that would need to be traversed to place a needle to reach the pleural space. The acquisition of the depth to the pleural space is hypothesized to allow for a higher rate of procedural success, given a location that is most appropriate for the available NT catheter would be chosen.

Similar to previous studies in other environments, non-ultrasound trained participants, the prehospital ALS providers in our example, quickly learned to apply ultrasound in the detection and diagnosis of pneumothorax in both cinematic and M-mode scenarios when tested with visual stimulus following the session.

These results add to the growing body of evidence that compact, handheld ultrasound devices can be utilized by prehospital personnel to potentially improve safety and effectiveness during high-risk, low-volume skills when compared to traditional techniques. These devices have been previously shown to be accurate in the acquisition of images compared to traditional non-handheld devices [21], which demonstrates the potential feasibility of implementing an EMS agency deploying such a device in the field to assist in procedures such as needle thoracostomy.

Future studies should focus on utilizing handheld ultrasound devices within a clinical setting, either in a prospective cohort or randomized control setting to determine if the use of such devices enhances safety and patient care in various EMS environments, including rural, suburban, urban, and helicopter settings. We would also encourage the expansion of the research setting to include both North American and European model EMS systems. This would build on the current study to allow for more generalizability from the non-clinical simulation environment seen here and the often austere environment encountered by prehospital paramedics and nurses when performing this skill in real-time. Further research would also benefit from a focus on the addition of comparison arms to validate the intervention, including in-service sessions on the treatment of tension pneumothorax with needle thoracostomy without ultrasound teaching or ultrasound devices. Additionally, our study was focused on process and disease-oriented outcomes. The addition of patient-oriented outcomes would help strengthen future studies.

In summary, within this cohort of rural North American EMS paramedics and nurses, a brief educational intervention involving teaching-focused thoracic ultrasound was feasible to deliver. Ultrasound guidance required an average of ten seconds longer, but increased safe and accurate simulated needle thoracostomy placement with fewer identified potential iatrogenic injuries. Further research is necessary to confirm these findings in a clinical setting. Our study adds to a growing body of evidence suggesting that prehospital ultrasound for needle thoracostomy may be safe and effective.

## Supplementary Information

**Additional file 1: **Data collection tool.

## Data Availability

The datasets used and/or analyzed during the current study are available from the corresponding author on reasonable request.

## Abbreviations

ALS: Advanced life support
BMI: Body mass index
, EMS: Emergency medical services
EMT-P: Emergency Medical Technician—Paramedic
IRB: Institutional Review Board
NT: Needle thoracostomy
POCUS: Point-of-care ultrasound
PHRN: Prehospital registered nurse

## References

[1] Wernick B, Hon HH, Mubang RN (2015). Complications of needle thoracostomy: a comprehensive clinical review. Int J Crit Illn Inj Sci.

[2] Pentecost G, Coughenour J, Sampson C (2020). Misplaced needle thoracostomy. V J Emerg Med.

[3] Kaserer A, Stein P, Simmen HP (2017). Failure rate of prehospital chest decompression after severe thoracic trauma. Am J Emerg Med.

[4] Lesperance RN, Carroll CM, Aden JK (2018). Failure rate of prehospital needle decompression for tension pneumothorax in trauma patients. Am Surg.

[5] Ball CG, Wyrzykowski AD, Kirkpatrick AW, Dente CJ, Nicholas JM, Salomone JP, Rozycki GS, Kortbeek JB, Feliciano DV (2010). Thoracic needle decompression for tension pneumothorax: clinical correlation with catheter length. Can J Surg.

[6] Henry R, Ghafil C, Golden A (2021). Prehospital needle decompression improves clinical outcomes in helicopter evacuation patients with multisystem trauma: a multicenter study. J Spec Oper Med.

[7] Noble VE, Lamhaut L, Capp R (2009). Evaluation of a thoracic ultrasound training module for the detection of pneumothorax and pulmonary edema by prehospital physician care providers. BMC Med Educ.

[8] McNeil CR, McManus J, Mehta S (2009). The accuracy of portable ultrasonography to diagnose fractures in an austere environment. Prehosp Emerg Care.

[9] Khalil PA, Merelman A, Riccio J (2021). Randomized controlled trial of point-of-care ultrasound education for the recognition of tension pneumothorax by paramedics in prehospital simulation. Prehosp Disaster Med.

[10] Oliver P, Bannister P, Bootland D (2020). Diagnostic performance of prehospital ultrasound diagnosis for traumatic pneumothorax by a UK helicopter emergency medical service. Europ J Emerg Med.

[11] Lyon M, Walton P, Bhalla V, Shiver SA (2012). Ultrasound detection of the sliding lung sign by prehospital critical care providers. Am J Emerg Med.

[12] Lyon M, Shiver SA, Walton P (2012). M-mode ultrasound for the detection of pneumothorax during helicopter transport. Am J Emerg Med.

[13] Knudtson JL, Dort JM, Helmer SD, Smith RS (2004). Surgeon-performed ultrasound for pneumothorax in the trauma suite. J Trauma Acute Care Surg.

[14] Dulchavsky SA, Schwarz KL, Kirkpatrick AW (2001). Prospective evaluation of thoracic ultrasound in the detection of pneumothorax background: thoracic ultrasound. J Trauma.

[15] Wilkerson RG, Stone MB (2010). Sensitivity of bedside ultrasound and supine anteroposterior chest radiographs for the identification of pneumothorax after blunt trauma. Acad Emerg Med..

[16] van der Weide L, Popal Z, Terra M, Schwarte LA, Ket JCF, Kooij FO, Exadaktylos AK, Zuidema WP, Giannakopoulos GF (2019). Prehospital ultrasound in the management of trauma patients: systematic review of the literature. Injury.

[17] El Zahran T, El Sayed MJ (2018). Prehospital ultrasound in trauma: a review of current and potential future clinical applications. J Emerg Trauma Shock.

[18] Taylor J, McLaughlin K, McRae A, Lang E, Anton A (2014). Use of prehospital ultrasound in North America: a survey of emergency medical services medical directors. BMC Emerg Med.

[19] Taylor LA, Vitto MJ, Joyce M (2018). Ultrasound-guided thoracostomy site identification in healthy volunteers. Crit Ultrasound J.

[20] Al-Shaqsi S (2010). Models of international emergency medical service (EMS) systems. Oman Med J.

[21] Dewar ZE, Wu J, Hughes H, Adnani A, Christiansen G, Ovedovitz L, Rittenberger JC (2020). A comparison of handheld ultrasound versus traditional ultrasound for acquisition of RUSH views in healthy volunteers. J Am Coll Emerg Phys Open.

