# Supplementary material for: Prehospital portable ultrasound for safe and accurate prehospital needle thoracostomy: a pilot educational study
Source: Ultrasound J. 2022 Jun 13;14:23. doi: 10.1186/s13089-022-00270-w (PMC9191400; doi:10.1186/s13089-022-00270-w)
Supplement: Supplementary file 1 — Additional file 1: Data collection tool. [file 13089_2022_270_MOESM1_ESM.docx]

# S1. Data Collection Tool

Data Collection Tool

Participant Number: _________

Gender: _________ Age: ____________ Years’ Experience: ___________

Certification Level: Paramedic PHRN PHPE PHP

Does Participant work for Critical Care Transport? Yes No

Previous Formal Ultrasound Training? Yes No

Pre-Teaching:

Anterior Needle Decompression Position:

Time to locate decompression site: ___________ (seconds) Located second intercostal space at mid-clavicular line? Yes No

If no, which intercostal space identified?

Dangerous underlying structure? Yes No

If yes, what structure: Vascular Heart Diaphragm Spleen Liver Other ________

Estimated Depth: ___________________ (cm)

Actual Depth: ___________________ (cm)

Lateral Needle Decompression Position:

Time to locate decompression site: ___________ (seconds)

Located 4^th^/5^th^ intercostal space at mid OR anterior axillary line? Yes No

If no, which intercostal space identified?

Dangerous underlying structure? Yes No

If yes, what structure: Vascular Heart Diaphragm Spleen Liver Other ________

Estimated Depth: ___________________ (cm)

Actual Depth: ___________________ (cm)

Post-Teaching with Ultrasound:

Anterior Needle Decompression Position:

Time to locate decompression site: ___________ (seconds) Located second intercostal space at mid-clavicular line? Yes No

If no, which intercostal space identified?

Dangerous underlying structure? Yes No

If yes, what structure: Vascular Heart Diaphragm Spleen Liver Other ________

Estimated Depth: ___________________ (cm)

Actual Depth: ___________________ (cm)

Lateral Needle Decompression Position:

Time to locate decompression site: ___________ (seconds)

Located 4th/5th intercostal space at mid OR anterior axillary line? Yes No

If no, which intercostal space identified?

Dangerous underlying structure? Yes No

If yes, what structure: Vascular Heart Diaphragm Spleen Liver Other ________

Estimated Depth: ___________________ (cm)

Actual Depth: ___________________ (cm)

Thoracic Ultrasound Clip Interpretation:

1. Interpretation: PTX No PTX Correct: Yes No

2. Interpretation: PTX No PTX Correct: Yes No

3. Interpretation: PTX No PTX Correct: Yes No

4. Interpretation: PTX No PTX Correct: Yes No

5. Interpretation: PTX No PTX Correct: Yes No

M-Mode Ultrasound Screenshot Interpretation:

6. Interpretation: PTX No PTX Correct: Yes No

7. Interpretation: PTX No PTX Correct: Yes No

8. Interpretation: PTX No PTX Correct: Yes No

9. Interpretation: PTX No PTX Correct: Yes No

10. Interpretation: PTX No PTX Correct: Yes No
